# A Case of Acute Ischemic Stroke Treated With Alteplase Immediately After Transcatheter Aortic Valve Implantation: Which Procedures or Surgeries are Considered Contraindications to Thrombolytics?

**DOI:** 10.7759/cureus.30136

**Published:** 2022-10-10

**Authors:** Rafik Mughnetsyan, Jamie Jacobs, April Dun, Prissilla Xu, Paul Vega, Sarkis Kiramijyan, Antonio K Liu

**Affiliations:** 1 Internal Medicine, Adventist Health White Memorial, Los Angeles, USA; 2 Cardiology, Adventist Health White Memorial, Los Angeles, USA; 3 Pharmacy Services, Adventist Health White Memorial, Los Angeles, USA; 4 Neurology, Adventist Health White Memorial, Los Angeles, USA; 5 Neurology, Loma Linda University School of Medicine, Loma Linda, USA

**Keywords:** transcatheter aortic valve implantation (tavi), contraindication, major surgery, alteplase, stroke

## Abstract

Undergoing a major surgery within 14 days is considered a contraindication for intravenous alteplase. However, there is no consensus as to what qualifies as major surgery or an invasive procedure. Occasionally, determining whether a procedure is "invasive" or too risky in the setting of emergency ischemic stroke thrombolytic management can be challenging. Stroke neurologists may not be able to make such a decision on their own. Guidance or clearance from the physicians who performed the procedure is essential. We report the case of a patient who received intravenous alteplase after developing a stroke immediately following transcatheter aortic valve implantation (TAVI).

## Introduction

Transcatheter aortic valve implantation (TAVI) is a minimally invasive, catheter-based approach to treating severe aortic stenosis. This therapy allows for a new aortic valve to be placed without traditional open-heart surgery. While the approach for the majority of TAVI procedures involves the femoral artery, transaortic, transapical, and even carotid artery approaches are being employed [1]. Femoral artery delivery requires the use of a 14-18 French sheath to be placed at the femoral artery site, which is typically sealed with a suture-mediated closure device at the completion of the procedure. The procedure is typically performed in the cardiac catheterization lab, under moderate sedation or general anesthesia. Acute ischemic stroke is a known complication after TAVI. The risk is highest among patients with atrial fibrillation not on anticoagulants in the first 90 days after TAVI [2]. While successful use of intravenous alteplase after TAVI has been described [3], it is infrequent. Hence alteplase’s safety after TAVI is not well established.

Intravenous alteplase has long been established as the standard of care for acute stroke with an onset of less than 4.5 hours. Among the exclusion criteria, no major surgery within the past 14 days and recent arterial puncture at non-compressible sites entail relative contraindications [4]. Usually, the presence of such contraindications is obvious and well agreed upon. However, when encountering newer, less frequent, and invasive procedures, the decision is often less obvious. In determining whether a procedure is "major" or invasive, full knowledge of the procedure itself is required. In an emergency setting, the treating neurologist may not have the knowledge or the time to gain this knowledge. In such a scenario, the physician who performed the procedure will have a major role to play in the treatment and outcome. We present the case of a patient who suffered an ischemic stroke immediately after TAVI and whose symptoms improved upon the uneventful administration of intravenous alteplase.

## Case presentation

**Figure 1:**
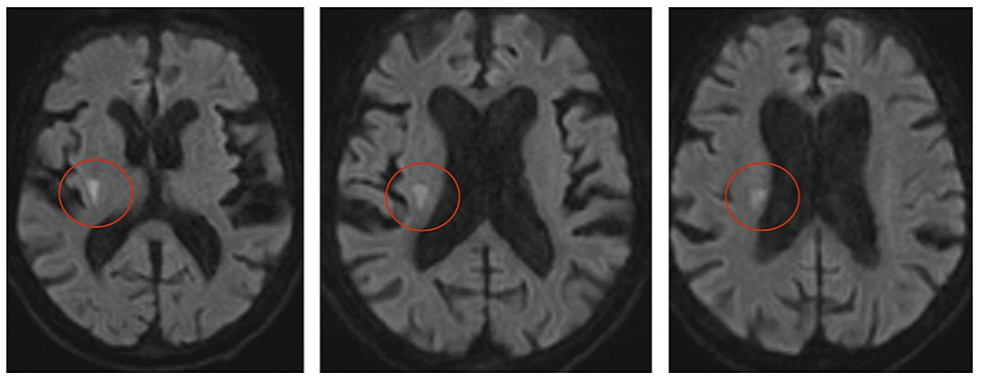
Brain MRI DWI sequence showing acute ischemic infarct (red circles) starting in basal ganglia and extending up to the right coronal radiata MRI: magnetic resonance imaging; DWI: diffusion-weighted imaging

The patient was a 91-year-old female with a significant history of hypertension and critical aortic stenosis. On the echocardiogram, the aortic valve area was 0.49 cm^2^, the mean gradient was 46.31 mmHg, the peak velocity was 4 m/s, the ejection fraction was 55%, and there was no intraventricular thrombus. The patient had an elective TAVI and was immediately extubated in the recovery room. Thirty minutes afterward, nurses noticed a sudden-onset left-sided weakness, and a stroke alert was initiated. On examination, the patient had significant weakness and neglect of the left side. The National Institutes of Health Stroke Scale (NIHSS) score was 10 (range: 0-42, with a score of 5-15 representing moderate stroke). Head CT was negative with no evidence of bleeding or air embolization. Her head MRI had already shown a developing acute ischemic infarct in the right basal ganglia extending into the corona radiata (Figure 1). Besides the fact that the patient had just undergone a TAVI via a trans-femoral approach, there were no other exclusion criteria to be considered. A long, detailed discussion between the neurologist and the interventional cardiologist led to the conclusion that the benefit of the potential reversal of ischemia by alteplase outweighed the risk of bleeding after TAVI. Furthermore, the patient had been a healthy, independent, and high-functioning individual prior, and a dense left hemiparesis would have been devastating and disabling. After obtaining proper consent, intravenous alteplase was administered. The patient responded well with improved symptoms and there was no alteplase-related complication. The patient was eventually discharged from the hospital with improved neurological status; the NIHSS score on discharge was 2.

## Discussion

According to one study, stroke is estimated to complicate 2.7-5.5% of TAVI by day 30 with most of them occurring within the first 48 hours [4]. The indication for alteplase was well established in our case. The patient experienced a sudden-onset focal neurologic deficit of moderate severity within the first 4.5 hours of the treatment window. The head CT had ruled out a hemorrhage (an absolute contraindication) as well as the presence of air within the brain parenchyma (a documented side effect of TAVI) [5]. Air is identified as very low-attenuation foci on CT and absent signal on MRI; neither one of them was present [6]. The remaining concern involved the potential risk posed by TAVI. The exact wording in the 2019 guideline for the early management of patients with acute ischemic stroke table 6 is as follows: "use of IV alteplase in carefully selected patients presenting in acute ischemic stroke who have undergone a major surgery in the preceding 14 days may be considered, but the potential increased risk of surgical-site hemorrhage should be weighed against the anticipated benefits of reduced stroke-related neurological deficits" [7]. Wordings like "carefully selected", "major", "may be considered" and "potential" are well-considered phrases put together after a detailed review of the literature and validated by many stroke experts. However, interpretation may be required, and going through such an exercise usually takes time and should be engaged in well before a real stroke alert.

In a litigation-rich environment, reporting a complication takes effort, courage, time (before the expiration of stature of limitation), and commitment. Bleeding complications are believed to be under-reported. Therefore, one may never know the real incidence of bleeding complications of alteplase use after TAVI or any other procedures. The 2019 practice guidelines hardly contain any actual published bleeding complication data to draw conclusions from [3,7,8]. Nevertheless, there are certain studies that help to define what constitutes a major surgery and the high risk of post-operation bleeding. An article in Stroke addressed thrombolysis in postoperative stroke in 2017 [9]; the authors admitted that the definition of minor versus major surgery remained controversial. A surgery is considered "major" if it involves the opening of major body cavities (abdomen, chest, or skull), is done in well-vascularized tissues or large arteries, is done by a team of doctors using general anesthesia, or requires a stay of at least one night in the hospital. This piece of literature is also quoted by the European Stroke Organisation guidelines on intravenous thrombolysis in their determination of what constitutes a major surgery [10].

Unable to find direct statements on the precise definition of major surgery, we also investigated two papers that discussed the off-label use of alteplase [11,12], but neither paper included cases of major surgery within 14 days. An article from 2001 has addressed the safety and efficacy of intraarterial thrombolysis for peri-cardiac operation stroke [13], but a search for literature addressing the safety of intravenous alteplase after a procedure that involved the aorta and major valve revealed no available data so far. 

Risk stratification occurs at the local expert level. For example, the University of California, Los Angeles has put forth a five-tier risk system [14]. Aortic, cardiac, intrathoracic, and transplant surgery belong to the very high-risk category. Without the clearance of the operating cardiologist, one can easily imagine a neurologist, who is usually not familiar with the details of TAVI, withholding treatment.

In our current case, all clinicians jointly reached the treatment decision after rapid sharing of medical, surgical, and technical information during the stroke alert. Intravenous alteplase was administered in a timely fashion and the patient's symptoms greatly improved with no complications.

## Conclusions

Intravenous alteplase has long been established as the standard of care for patients with ischemic strokes that fulfill the inclusion and exclusion criteria. However, major surgery or invasive procedure within 14 days remains a safety concern. A precise definition of what constitutes a major surgery or procedure is lacking and practice guidelines usually leave room for interpretation. As medical and surgical sciences advance, stroke presentation after novel procedures must be anticipated. Establishing alteplase's safety profile further requires rigorous reporting of thrombolytic use outcomes in stroke after TAVR and other novel procedures. Most important of all, the active participation of all consultants, patients, and family members involved is essential in optimizing the treatment and outcome of post-procedural stroke.
